# Oxidative Stress-Mediated Mitochondrial Dysfunction Drives Genistein-Induced Apoptosis in TM4 Sertoli and ELT3 Leiomyoma Cells

**DOI:** 10.3390/jox16050156

**Published:** 2026-08-22

**Authors:** Samak Sutjarit, Chainarong Sakulthaew, Nattakan Meekhanon, Kazuhiko Ochiai

**Affiliations:** 1 Department of Veterinary Nursing, Faculty of Veterinary Technology, Kasetsart University, Bangkok 10900, Thailand; cvtcns@ku.ac.th (C.S.); cvtnkl@ku.ac.th (N.M.); 2 School of Veterinary Nursing and Technology, Faculty of Veterinary Science, Nippon Veterinary and Life Science University, Tokyo 180-8602, Japan; kochiai@nvlu.ac.jp

**Keywords:** apoptosis, ELT3 cells, genistein, oxidative stress, TM4 cells

## Abstract

Genistein (GEN) exhibits concentration- and cell-type-dependent biological activities; however, the mechanisms underlying its cytotoxicity remain incompletely understood. This study investigated whether oxidative stress-mediated mitochondrial dysfunction contributes to GEN-induced apoptosis in TM4 mouse Sertoli cells and ELT3 rat leiomyoma cells. Cells were exposed to GEN (30 or 100 µM) for 48 h. Cytotoxicity, oxidative stress, mitochondrial function, and apoptosis were assessed by measuring cell viability, lactate dehydrogenase release, reactive oxygen species (ROS), malondialdehyde, glutathione reductase activity, mitochondrial membrane potential (ΔΨm), intracellular ATP, apoptosis-related gene expression, and caspase-3/-9 activities. The involvement of oxidative stress was examined using N-acetyl-L-cysteine (NAC). GEN reduced cell viability, antioxidant capacity, ΔΨm, and ATP content while increasing membrane damage, ROS accumulation, and lipid peroxidation in both cell lines. These changes were accompanied by upregulation of Bax, Tp53, caspase-3, and caspase-9; downregulation of Bcl-2; and increased caspase-3/-9 activities. NAC pretreatment attenuated these alterations, supporting ROS as an upstream mediator. Notably, TM4 cells were approximately 4.4-fold more sensitive to GEN than ELT3 cells at 48 h. Collectively, the findings support a ROS-dependent mitochondrial apoptotic response under elevated in vitro GEN exposure but do not establish selective antitumor cytotoxicity or direct physiological relevance.

## 1. Introduction

Nutraceuticals have gained considerable attention as alternatives or complements to conventional pharmaceuticals because they provide medicinal and physiological benefits beyond basic nutritional value [1,2]. The increasing global consumption of nutraceutical products has intensified interest in understanding not only their beneficial biological activities but also the molecular mechanisms underlying their health effects. Among plant-derived bioactive compounds, flavonoids constitute a diverse class of naturally occurring polyphenolic phytochemicals that have been extensively investigated because of their broad pharmacological properties, including antioxidant, anti-inflammatory, and anticancer activities [3,4,5].

Flavonoids are widely distributed in fruits, vegetables, seeds, and other plant-derived foods, where they contribute to a variety of physiological processes in both plants and animals [6]. One of the most extensively studied flavonoids is genistein (GEN), a naturally occurring isoflavone predominantly found in soybeans, soy-based foods, nuts, and legumes [7]. Owing to its structural similarity to 17β-estradiol, GEN interacts with both estrogen receptor α (ERα) and estrogen receptor β (ERβ), exhibiting a markedly higher binding affinity for ERβ. Through estrogen receptor-dependent and receptor-independent mechanisms, GEN regulates multiple signaling pathways involved in cell proliferation, differentiation, apoptosis, oxidative stress, and mitochondrial function. Notably, these biological activities are highly dependent on the cellular context, exposure concentration, and duration of treatment [8,9]. Numerous studies have demonstrated that GEN exhibits diverse biological activities, including antioxidant, anti-inflammatory, and anticancer effects [10,11]. In experimental models, GEN has been reported to suppress tumor growth through the modulation of estrogen receptor signaling, inhibition of cell migration, induction of cell-cycle arrest, and activation of apoptosis [12,13,14,15,16,17]. The antiproliferative activity of GEN has been documented in several cancer cell models, including human breast cancer and hepatocarcinoma cells, although the underlying responses appear to vary according to cellular and hormonal contexts [18,19]. Moreover, accumulating evidence indicates that the biological activity of GEN is highly context-dependent. At relatively low concentrations, GEN generally exerts antioxidant effects, whereas higher concentrations or prolonged exposure may promote reactive oxygen species (ROS) generation and oxidative injury [20]. Excessive ROS disrupt cellular redox homeostasis, leading to oxidative damage of lipids, proteins, and nucleic acids, ultimately resulting in mitochondrial dysfunction and cell death [21]. Because mitochondria serve as both a major source and a principal target of ROS, oxidative stress is closely associated with mitochondrial membrane depolarization, impaired ATP production, and activation of intrinsic apoptotic pathways [22]. Therefore, determining whether oxidative stress-mediated mitochondrial dysfunction represents a key mechanism underlying GEN-induced cytotoxicity is essential for understanding its biological effects. In addition to their well-established pharmacological properties, flavonoids have attracted increasing attention because of their potential influence on the reproductive system. Previous studies have shown that flavonoids and phytoestrogens can modulate reproductive physiology through interactions with endocrine signaling pathways and cellular redox homeostasis [23,24]. Given its estrogen-like activity, GEN has raised concerns regarding its potential effects on reproductive tissues. Although GEN-induced apoptosis has been reported in several cell types, whether oxidative stress acts as the primary upstream event leading to mitochondrial dysfunction in reproductive cells remains incompletely understood. This question is particularly relevant for Sertoli cells, which are highly specialized somatic cells within the seminiferous tubules that provide structural and metabolic support for developing germ cells, maintain the blood–testis barrier, and regulate signaling pathways essential for normal spermatogenesis and male fertility [25].

To address this knowledge gap, the present study employed two biologically distinct reproductive cell models. Mouse Sertoli (TM4) cells were selected as a representative model of normal male reproductive support cells because of their indispensable role in spermatogenesis and their high sensitivity to oxidative stress. In contrast, Eker leiomyoma tumor-3 (ELT3) cells represent a tumor-derived reproductive cell model characterized by altered metabolic activity and proliferative capacity and are widely used to investigate oxidative stress, mitochondrial dysfunction, and chemically induced cellular injury [26,27]. Comparative evaluation of TM4 and ELT3 cells therefore enables the assessment of whether oxidative stress-mediated mitochondrial dysfunction represents a conserved mechanism of GEN-induced cytotoxicity while simultaneously identifying cell-type-specific differences in susceptibility to oxidative injury.

Although GEN is widely consumed through soy-based diets, circulating plasma concentrations in humans are generally within the low micromolar range (approximately 0.1–5 µM), depending on dietary intake and metabolic status [28]. Nevertheless, intracellular or tissue-specific concentrations may become substantially higher because of tissue accumulation, repeated exposure, or altered metabolism. Consequently, in vitro studies commonly employ higher concentrations to characterize dose-dependent cellular responses and elucidate the molecular mechanisms underlying toxicological effects.

Based on these considerations, we hypothesized that GEN induces oxidative stress, leading to mitochondrial dysfunction and activation of the intrinsic apoptotic pathway in both TM4 and ELT3 cells, although the magnitude of these responses may differ according to cell type. Therefore, the present study investigated whether GEN induces a conserved oxidative stress-mediated mitochondrial apoptotic pathway across these biologically distinct reproductive cell models. By comparing normal male reproductive support cells with tumor-derived reproductive cells, this study aimed to identify both shared and cell-type-specific mechanisms of GEN-induced cytotoxicity. The findings are expected to provide new mechanistic insight into the role of oxidative stress in GEN-induced mitochondrial dysfunction and apoptosis and contribute to a more comprehensive evaluation of the biological effects and safety of this widely consumed nutraceutical flavonoid.

## 2. Materials and Methods

### 2.1. Cells and Chemicals

Genistein (GEN; ≥98% purity), N-acetylcysteine (NAC; ≥99% purity), and dimethyl sulfoxide (DMSO; cell culture grade) were purchased from Sigma-Aldrich (St. Louis, MO, USA). TM4 and ELT3 cells were obtained from the American Type Culture Collection (ATCC) (Manassas, VA, USA). All assay kits used in this study were obtained from Abcam (Cambridge, UK), including the MTS Cell Proliferation Assay Kit (ab197010), LDH Cytotoxicity Assay Kit II (ab65393), DCFDA ROS Detection Kit (ab113851), Lipid Peroxidation (MDA) Assay Kit (ab118970), Glutathione Reductase (GR) Assay Kit (ab83461), CellTiter-Glo^®^ Luminescent Cell Viability Assay (Promega Corporation, Madison, WI, USA), Caspase-3 Activity Assay Kit (ab39401), Caspase-9 Activity Assay Kit (ab65608), and Pierce™ BCA Protein Assay Kits (Thermo Fisher Scientific, Waltham, MA, USA). All assays were performed strictly according to the manufacturers’ instructions. Cell culture reagents, including DMEM/F-12 medium, fetal bovine serum (FBS), horse serum, penicillin, and streptomycin, were purchased from Gibco (Thermo Fisher Scientific, USA).

### 2.2. Cell Culture and Assays

TM4 and ELT3 cells were cultured in complete DMEM/F-12 supplemented with 5% horse serum, 2.5% FBS, 100 U/mL penicillin, and 100 U/mL streptomycin in a humidified atmosphere with 5% CO_2_ at 37 °C. Cell viability, intracellular ROS production, lipid peroxidation, and glutathione reductase activity were determined using protocols previously established in our laboratory and optimized for the present study [29].

### 2.3. Determination of Mitochondrial Membrane Potential (ΔΨm)

ΔΨm was determined using the JC-1 fluorescent probe and quantified with a fluorescence microplate reader. Cells were seeded in black 96-well plates with clear bottoms and allowed to be attached overnight, after which they were treated with GEN at concentrations of 30 and 100 μM for 48 h; control cells received the vehicle only. Following treatment, the culture medium was removed, and cells were incubated with JC-1 dye (5 μg/mL) diluted in serum-free culture medium at 37 °C for 20 min in the dark. After incubation, the cells were gently washed twice with warm phosphate-buffered saline (PBS) to remove excess dye, and fresh PBS was added to each well. Fluorescence intensity was measured using a fluorescence microplate reader at excitation/emission wavelengths of 485/530 nm for JC-1 monomers (green fluorescence) and 535/590 nm for JC-1 aggregates (red fluorescence). Mitochondrial membrane potential was expressed as the ratio of red to green fluorescence intensity. Each experiment was performed in triplicate and repeated independently at least three times. To validate the sensitivity of the JC-1 assay, a subset of cells was treated with carbonyl cyanide m-chlorophenyl hydrazone (CCCP; 10 μM) for 30 min prior to JC-1 staining as a positive control for mitochondrial membrane depolarization.

### 2.4. Determination of Intracellular ATP Levels

Intracellular ATP content was measured using a luminescence-based ATP assay. TM4 and ELT3 cells were seeded in white 96-well plates and exposed to GEN at 30 or 100 µM for 48 h. Following treatment, an equal volume of ATP detection reagent was added directly to each well, and the plates were shaken for 2 min to induce cell lysis. The plates were subsequently incubated at room temperature for 10 min to stabilize the luminescent signal. Luminescence was measured using a microplate reader, and the background signal was subtracted from each measurement. Total protein content was determined in corresponding parallel wells subjected to identical experimental conditions using the BCA protein assay. The background-corrected ATP luminescence was normalized to total protein content and expressed as relative luminescence units per milligram of protein (RLU/mg protein). For graphical presentation, the normalized values were additionally expressed relative to the corresponding control group, which was defined as 100%. Measurements were obtained from three independent experiments.

### 2.5. Determination of N-Acetylcysteine (NAC) Rescue Activity

To determine whether oxidative stress is a key mediator of GEN-induced cytotoxicity, TM4 and ELT3 cells were pretreated with NAC before GEN exposure. NAC was dissolved in sterile distilled water to prepare a stock solution and diluted in culture medium immediately before use. Cells were pretreated with 5 mM NAC for 1 h; subsequently, GEN (30 or 100 µM) was added directly to the NAC-containing culture medium without washing or replacing the medium, and the cells were co-incubated with NAC and GEN for an additional 48 h. Thus, NAC remained present throughout the entire GEN exposure period. The experimental groups consisted of the control, NAC alone, GEN 30 µM, GEN 100 µM, NAC + GEN 30 µM, and NAC + GEN 100 µM. Following treatment, cell viability, LDH release, intracellular ROS production, MDA levels, GR activity, ΔΨm, and intracellular ATP content were evaluated using the same assays described above. Data were obtained from at least three independent experiments and are presented as the mean ± SD.

### 2.6. Caspase Activity Assays

Caspase-3 and caspase-9 activities were measured using colorimetric assay kits based on cleavage of synthetic peptide substrates conjugated to p-nitroaniline (pNA). Active caspases cleave the substrates DEVD-pNA and LEHD-pNA for caspase-3 and -9, respectively, releasing free pNA, which produces a yellow chromophore detectable at 405 nm. The absorbance intensity is directly proportional to the caspase activity. Following treatment, TM4 and ELT3 cells were harvested and washed twice with cold PBS. Cells were lysed in chilled lysis buffer containing protease inhibitors and incubated on ice for 10–15 min. Lysates were centrifuged at 12,000× *g* for 10 min at 4 °C to remove debris, and the supernatant was collected for analysis. Protein concentration was determined using the BCA assay (Thermo Fisher Scientific), and all samples were normalized to equal protein concentrations. For caspase activity measurement, equal amounts of protein lysate were incubated with reaction buffer containing dithiothreitol (DTT, 10 mM) and the caspase-3 substrate DEVD-pNA and caspase-9 substrate LEHD-pNA (200 µM final concentration), in a total volume of 100 µL per each. Samples were incubated at 37 °C for 1–2 h in the dark. Absorbance was measured at 405 nm using a microplate reader. Background absorbance from blank wells (no lysate) was subtracted prior to analysis.

### 2.7. Quantitative Real-Time PCR (qPCR)

Total RNA was extracted from TM4 and ELT3 cells using TRIzol reagent (Invitrogen, Waltham, MA, USA), according to the manufacturer’s instructions. Briefly, after treatment, cells were washed twice with ice-cold PBS and lysed directly in TRIzol. Chloroform was added, and the samples were vigorously mixed and centrifuged at 12,000× *g* for 15 min at 4 °C to separate the aqueous phase. RNA was precipitated with isopropanol, washed with 75% ethanol, air-dried, and dissolved in RNase-free water [30]. RNA concentration and purity were determined spectrophotometrically using a NanoDrop spectrophotometer (Thermo Fisher Scientific, USA). Only RNA samples with an A260/A280 ratio between 1.8 and 2.0 were used for further analysis, and RNA integrity was additionally verified via agarose gel electrophoresis. For cDNA synthesis, 1 µg of total RNA was reverse transcribed using a cDNA synthesis kit (Thermo Fisher Scientific, USA) according to the manufacturer’s protocol. The reverse transcription reaction was performed in a total volume of 20 µL containing the RNA template, reverse transcriptase, reaction buffer, dNTP mixture, random primers, and RNase inhibitor. The reaction conditions were 25 °C for 10 min, 42 °C for 30 min, and 85 °C for 5 min to terminate the reaction. qPCR was carried out using SYBR Green Master Mix (Applied Biosystems, USA) on a real-time PCR system (Applied Biosystems, USA). Each PCR reaction was prepared in a total volume of 20 µL, containing 10 µL of SYBR Green Master Mix, 0.4 µL of forward primer (10 µM), 0.4 µL of reverse primer (10 µM), 2 µL of cDNA template, and 7.2 µL of nuclease-free water. Separate species-specific primer sets were used for the TM4 and ELT3 cells—mouse-specific primers were used for TM4 samples, whereas rat-specific primers were used for ELT3 samples. The corresponding reference accession numbers, primer sequences, and amplicon sizes are presented in Table 1. The qPCR cycling conditions were as follows: initial denaturation at 95 °C for 10 min, followed by 40 cycles of denaturation at 95 °C for 15 s, annealing at 58–60 °C for 30 s, and extension at 72 °C for 30 s. At the end of the amplification, a melt curve analysis was performed to confirm the specificity of the PCR products. Only samples showing a single sharp peak were included in the analysis. Each sample was analyzed in triplicate, and no-template controls were included in each run to rule out contamination. Relative gene expression levels were normalized to the housekeeping gene GAPDH and calculated using the 2-ΔΔCt method; the results were expressed as the fold change relative to the control group. The stability of GAPDH as the reference gene was assessed by comparing its raw Cq values across the control and GEN-treated groups. The analysis was performed separately for TM4 and ELT3 cells using one-way ANOVA followed by Tukey’s multiple-comparisons test.

### 2.8. Statistical Analysis

All experiments were performed using at least three independent biological replicates. Data normality was assessed using the Shapiro–Wilk test. For the MTS cell viability assay, data were analyzed using two-way analysis of variance (ANOVA), with GEN concentration and exposure time as fixed factors. The interaction between concentration and time was also evaluated. When significant main or interaction effects were detected, Tukey’s multiple-comparisons test was applied. For all other assays, including LDH release, ROS production, MDA content, GR activity, ΔΨm, intracellular ATP levels, caspase-3 and caspase-9 activities, and quantitative real-time PCR analyses, statistical differences among treatment groups were analyzed using one-way ANOVA followed by Bonferroni’s post hoc test. Data are presented as the mean ± standard deviation (SD). Differences were considered statistically significant at *p* < 0.05. All statistical analyses were performed using STATA version 17.0 (StataCorp LLC, College Station, TX, USA).

## 3. Results

### 3.1. GEN Decreased Cell Viability and Increased LDH Activity in TM4 and ELT3 Cells

**Figure 1:**
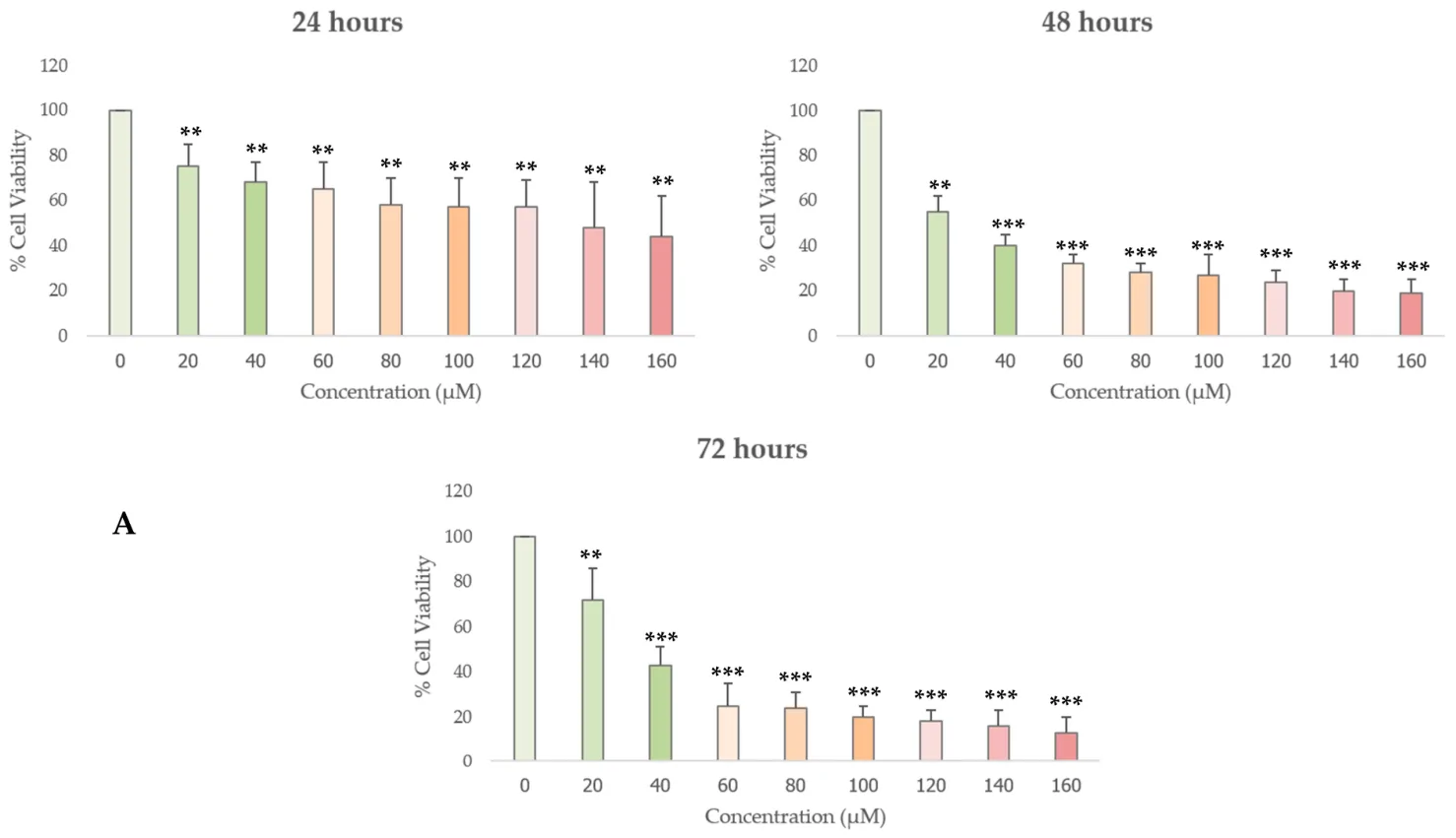

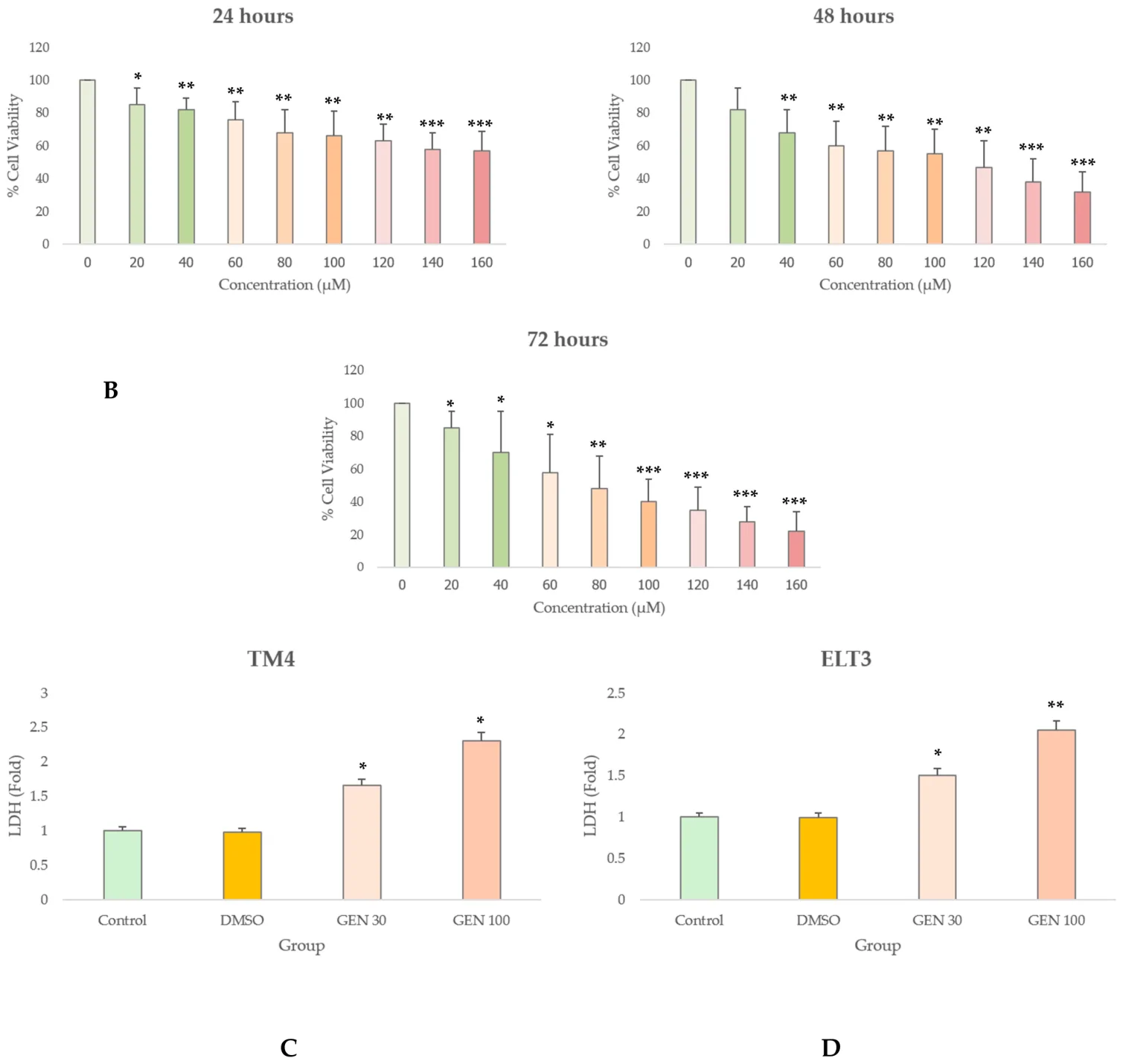
Effects of GEN on cell viability and LDH activity in TM4 and ELT3 cells. TM4 cells (**A**,**C**) and ELT3 cells (**B**,**D**) were treated with increasing concentrations of GEN (0–160 µM) for 24, 48, and 72 h for cell viability analysis, or with GEN (30 and 100 µM) for the indicated period for LDH activity analysis. Cell viability was determined using an MTS assay and expressed as percentage of cell viability, whereas LDH activity was measured as an indicator of membrane damage and expressed as fold change relative to control. Data are presented as mean ± SD from three independent experiments. Statistical significance was determined compared with control or between indicated groups (* *p* < 0.05, ** *p* < 0.01, *** *p* < 0.001).

GEN was evaluated for its growth-inhibitory effects on TM4 and ELT3 cells using the MTS assay. The half-maximal inhibitory concentration (IC_50_) values for TM4 cells were 131.0, 30.1, and 29.7 µM after 24, 48, and 72 h of exposure, respectively, whereas the corresponding IC_50_ values for ELT3 cells were 187.0, 132.5, and 90.5 µM. The progressive decrease in IC_50_ values over time indicates a time-dependent cytotoxic effect of GEN. At 48 h, the IC_50_ value of GEN was 30.1 µM in TM4 cells and 132.5 µM in ELT3 cells. Thus, TM4 cells exhibited approximately 4.4-fold greater sensitivity to GEN-induced loss of viability than ELT3 cells. This marked difference indicates substantial cell-type-dependent susceptibility, with the Sertoli cell model responding to GEN at considerably lower concentrations than the tumor-derived leiomyoma cell model. Therefore, 48 h was selected as the exposure period for all subsequent experiments because it produced measurable cytotoxicity while avoiding prolonged incubation. For mechanistic studies, common GEN concentrations of 30 and 100 µM were applied to both cell lines to enable direct comparison of oxidative stress, mitochondrial dysfunction, and apoptotic responses under identical experimental conditions. The 30 µM concentration was selected as a biologically relevant low dose based on the preliminary dose–response analysis and previous studies demonstrating measurable cellular responses without excessive loss of viability, whereas 100 µM was selected as a high-dose condition to evaluate concentration-dependent effects; therefore, GEN concentrations of 30 and 100 µM were selected for subsequent experiments. GEN significantly reduced the viability of TM4 and ELT3 cells in both concentration- and time-dependent manners (Figure 1A,B). After 24 h of exposure, moderate reductions in cell viability were observed at higher GEN concentrations, with progressively greater decreases detected at longer exposure periods and increased concentrations. These findings indicate that GEN exerts cytotoxic effects in both TM4 and ELT3 cells. To further evaluate membrane damage induced by GEN, LDH activity was measured in both cell lines. In TM4 cells (Figure 1C), GEN treatment significantly increased LDH release in a concentration-dependent manner. Exposure to 30 µM GEN significantly elevated LDH activity compared with the control group, indicating increased membrane permeability, while 100 µM GEN caused a more pronounced increase, reflecting enhanced cytotoxicity. DMSO treatment did not significantly alter LDH activity, confirming that the solvent had no detectable cytotoxic effect. Similarly, ELT3 cells (Figure 1D) exhibited significant concentration-dependent increases in LDH activity following GEN treatment. Both 30 and 100 µM GEN significantly increased LDH release compared with the control, with the highest LDH activity observed at 100 µM GEN. Notably, the magnitude of LDH elevation appeared slightly greater in ELT3 cells at the higher concentration. Overall, these findings demonstrate that GEN decreases cell viability and induces membrane damage in TM4 and ELT3 cells in a dose-dependent manner.

### 3.2. GEN Increased ROS and MDA Levels While Decreasing GR Activity in TM4 and ELT3 Cells

**Figure 2:**
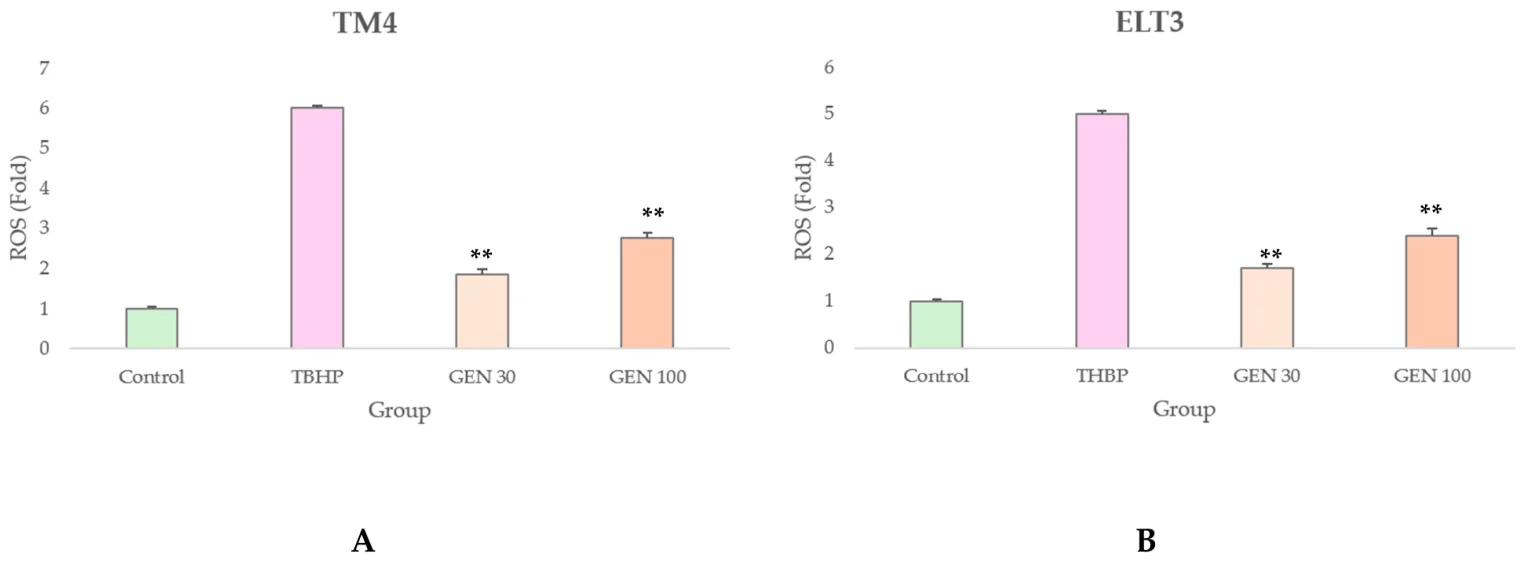

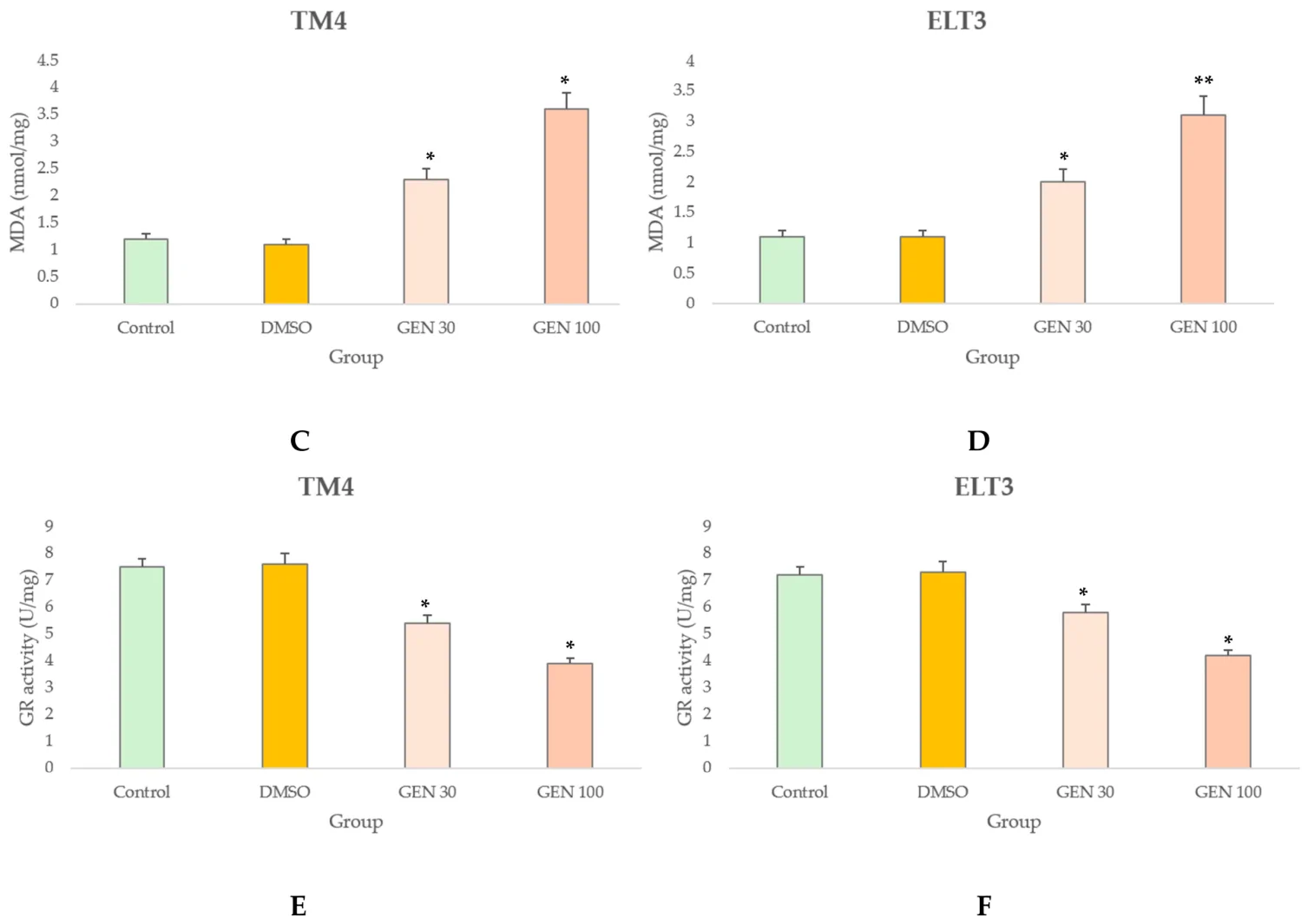
Effects of GEN on oxidative stress and antioxidant status in TM4 and ELT3 cells. TM4 cells (**A**,**C**,**E**) and ELT3 cells (**B**,**D**,**F**) were treated with GEN (30 and 100 µM). Intracellular ROS generation was determined using the DCFDA fluorescence assay and expressed relative to the control, while lipid peroxidation was evaluated by measuring MDA levels. GR activity was assessed as an indicator of cellular antioxidant defense and expressed as enzymatic activity. TBHP was used as a positive control for oxidative stress. Data are presented as mean ± SD from three independent experiments. Statistical significance was determined compared with control or between indicated groups (* *p* < 0.05, ** *p* < 0.01).

In TM4 cells (Figure 2A), GEN treatment significantly increased intracellular ROS production in a concentration-dependent manner. Exposure to 30 µM GEN caused a moderate but significant elevation in ROS levels compared with the control group, whereas 100 µM GEN produced a markedly greater increase. Treatment with TBHP, used as a positive control, resulted in the highest ROS accumulation, confirming effective induction of oxidative stress and validating assay responsiveness. Similarly, ELT3 cells (Figure 2B) demonstrated significantly increased ROS generation following GEN exposure. Both 30 µM and 100 µM GEN significantly elevated ROS levels compared with the control, with the greatest increase observed at the higher concentration, while TBHP again induced the strongest ROS production. Overall, these findings indicate that GEN enhances intracellular ROS generation in both TM4 and ELT3 cells in a dose-dependent manner. To determine whether increased ROS production was associated with oxidative membrane damage, lipid peroxidation was evaluated by measuring MDA levels. In TM4 cells (Figure 2C), GEN treatment significantly increased MDA levels compared with the control. Exposure to 30 µM GEN resulted in a moderate elevation in MDA, whereas 100 µM GEN caused a more pronounced increase, indicating concentration-dependent lipid peroxidation. Similarly, ELT3 cells (Figure 2D) exhibited significantly increased MDA levels following GEN treatment. Both 30 µM and 100 µM GEN significantly elevated MDA levels compared with the control, with the highest level observed at 100 µM. The greater increase at the higher concentrations further supports dose-dependent oxidative membrane damage. Overall, GEN significantly enhanced lipid peroxidation in both TM4 and ELT3 cells. To further investigate the effect of GEN on antioxidant defense systems, GR activity was measured in TM4 and ELT3 cells. In TM4 cells (Figure 2E), GEN treatment significantly decreased GR activity compared with the control. Exposure to 30 µM GEN caused a moderate reduction in enzyme activity, whereas 100 µM GEN resulted in a marked decrease. DMSO treatment did not significantly alter GR activity, indicating that the vehicle had no detectable effect on antioxidant function. Likewise, ELT3 cells (Figure 2F) showed significantly reduced GR activity following GEN exposure. Both 30 µM and 100 µM GEN significantly suppressed GR activity relative to the control, with the strongest inhibition observed at 100 µM. Collectively, these findings demonstrate that GEN impairs antioxidant enzyme activity in both TM4 and ELT3 cells in a concentration-dependent manner.

### 3.3. GEN Decreased ΔΨM and Normalized Intracellular ATP Levels in TM4 and ELT3 Cells

**Figure 3:**
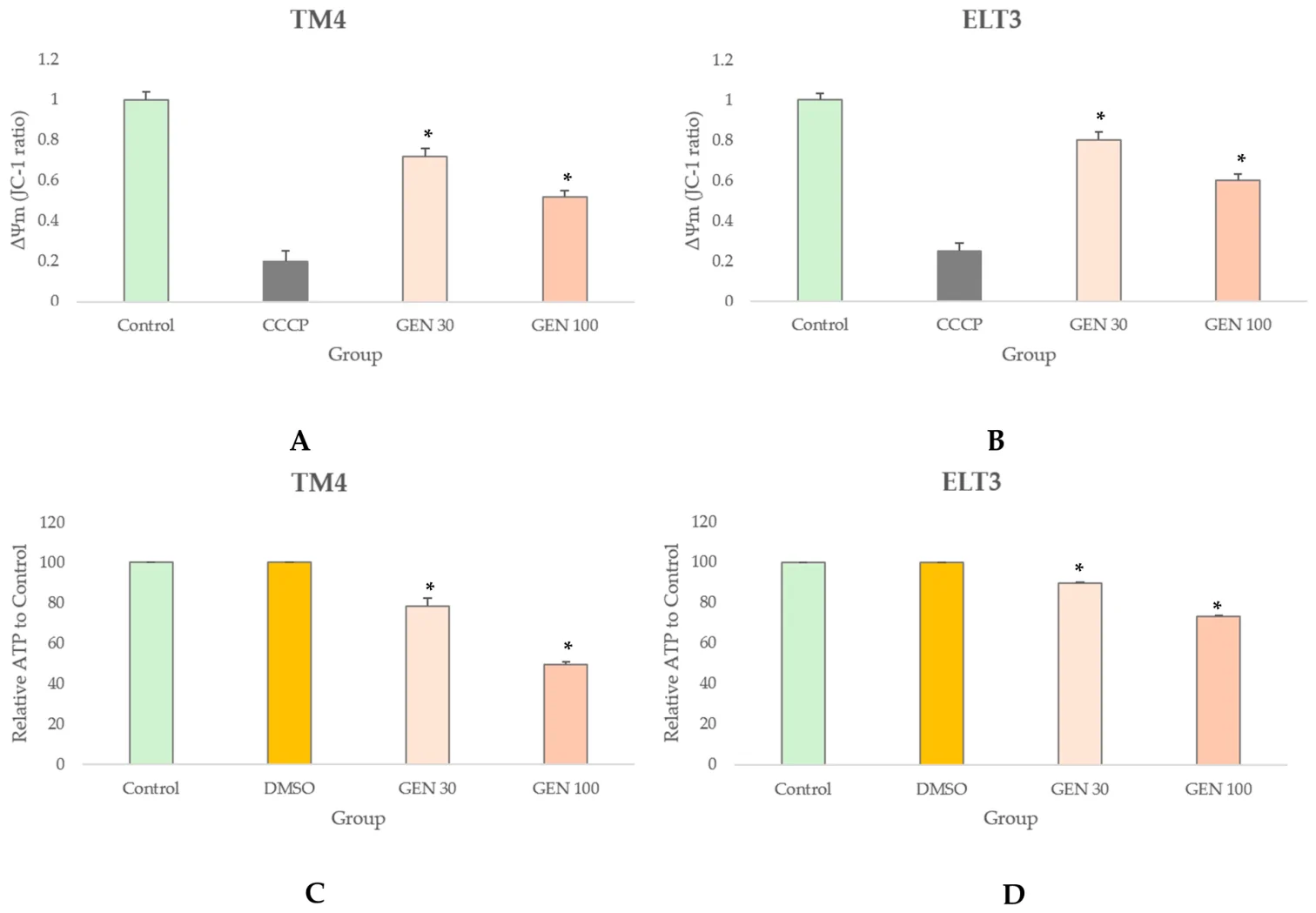
Effects of GEN on ΔΨm and normalized intracellular ATP levels in TM4 and ELT3 cells. TM4 and ELT3 cells were treated with GEN (30 or 100 µM for 48 h). ΔΨm was assessed using JC-1 staining and expressed as the red-to-green fluorescence ratio in (**A**) TM4 and (**B**) ELT3 cells. CCCP was included as a positive control for mitochondrial depolarization. Intracellular ATP levels were measured in (**C**) TM4 and (**D**) ELT3 cells. Background-corrected ATP luminescence was normalized to the total protein content of the corresponding samples and expressed as RLU/mg protein. Data are presented as mean ± SD from three independent experiments, with individual data points shown. * *p* < 0.05 versus the corresponding control group.

In TM4 cells (Figure 3A), GEN treatment significantly decreased ΔΨm in a concentration-dependent manner. Exposure to 30 µM GEN caused a moderate reduction in the JC-1 red/green fluorescence ratio compared with the control group, whereas 100 µM GEN produced a markedly greater decline. CCCP treatment resulted in the most pronounced loss of ΔΨm, confirming successful mitochondrial depolarization and validating the reliability of the assay. Likewise, ELT3 cells (Figure 3B) demonstrated significant mitochondrial depolarization following GEN treatment. Both 30 µM and 100 µM GEN significantly reduced ΔΨm relative to the control, with the strongest effect observed at 100 µM. CCCP again produced the greatest decrease in ΔΨm. Collectively, these findings indicate that GEN induces concentration-dependent mitochondrial dysfunction in both TM4 and ELT3 cells through disruption of mitochondrial membrane potential. To further investigate the effect of GEN on cellular energy metabolism, normalized intracellular ATP levels were measured in TM4 and ELT3 cells. GEN treatment significantly reduced normalized ATP levels in both TM4 (Figure 3C) and ELT3 cells (Figure 3D) compared with the control and DMSO-treated groups. The reduction in normalized ATP was more pronounced in TM4 cells than in ELT3 cells. In contrast, no significant difference was observed between the control and DMSO groups, indicating that the solvent itself had no detectable effect on ATP production. Overall, these results demonstrate that GEN decreases intracellular ATP levels in a concentration-dependent manner in both TM4 and ELT3 cells, suggesting impaired mitochondrial energy metabolism.

### 3.4. NAC Attenuates GEN-Induced Cell Viability and Oxidative Stress Markers in TM4 and ELT3 Cells

**Figure 4:**
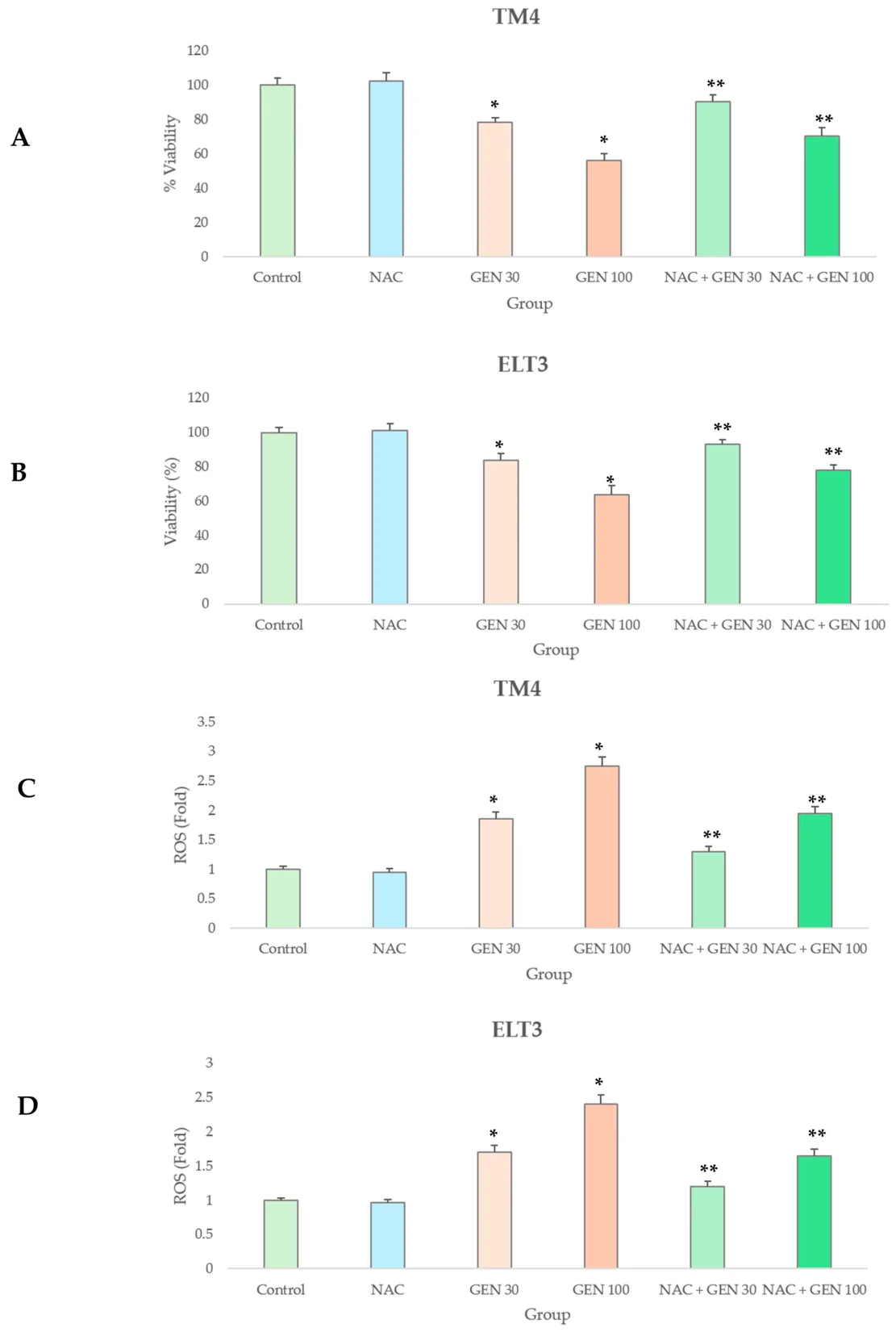

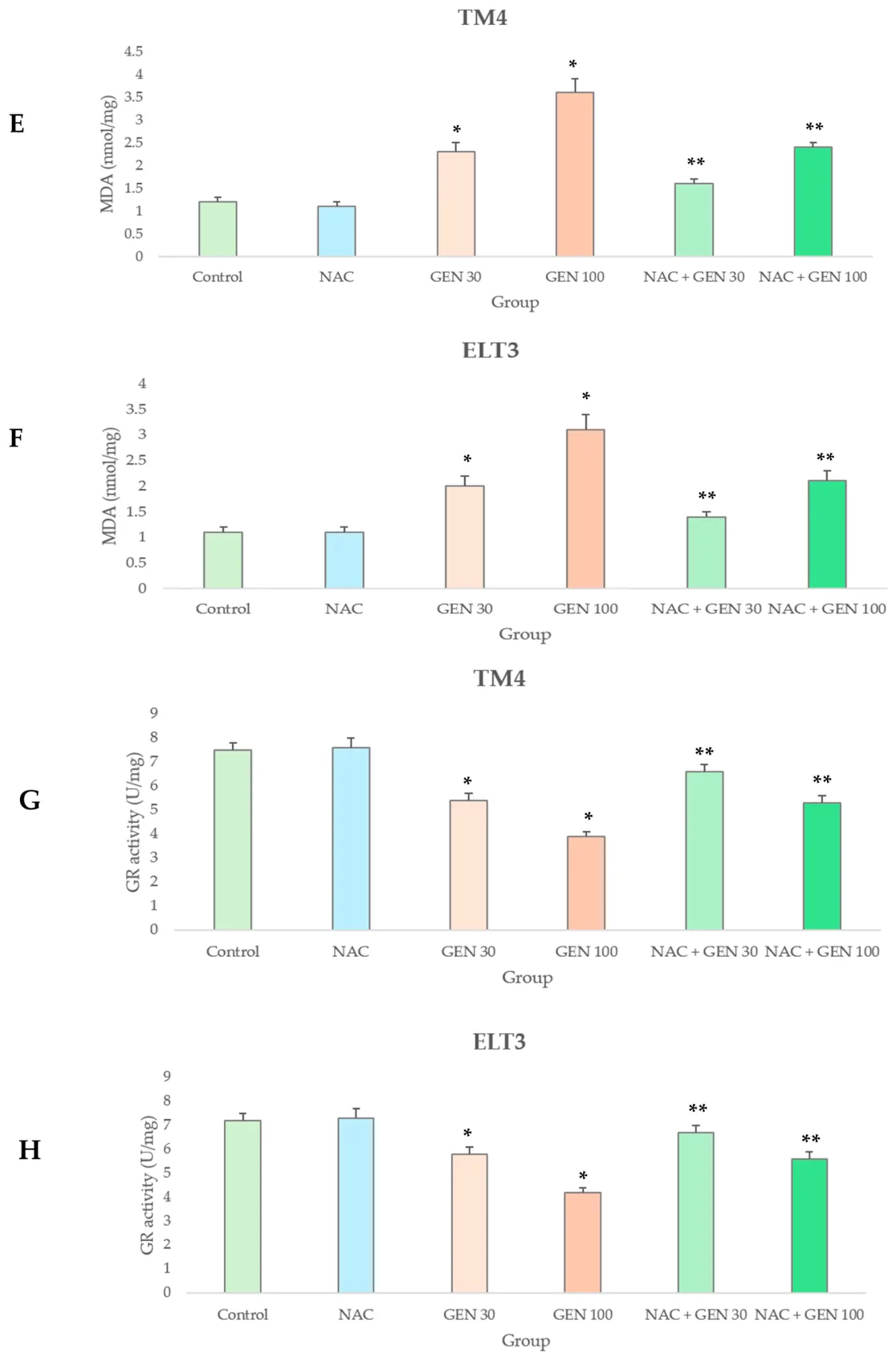

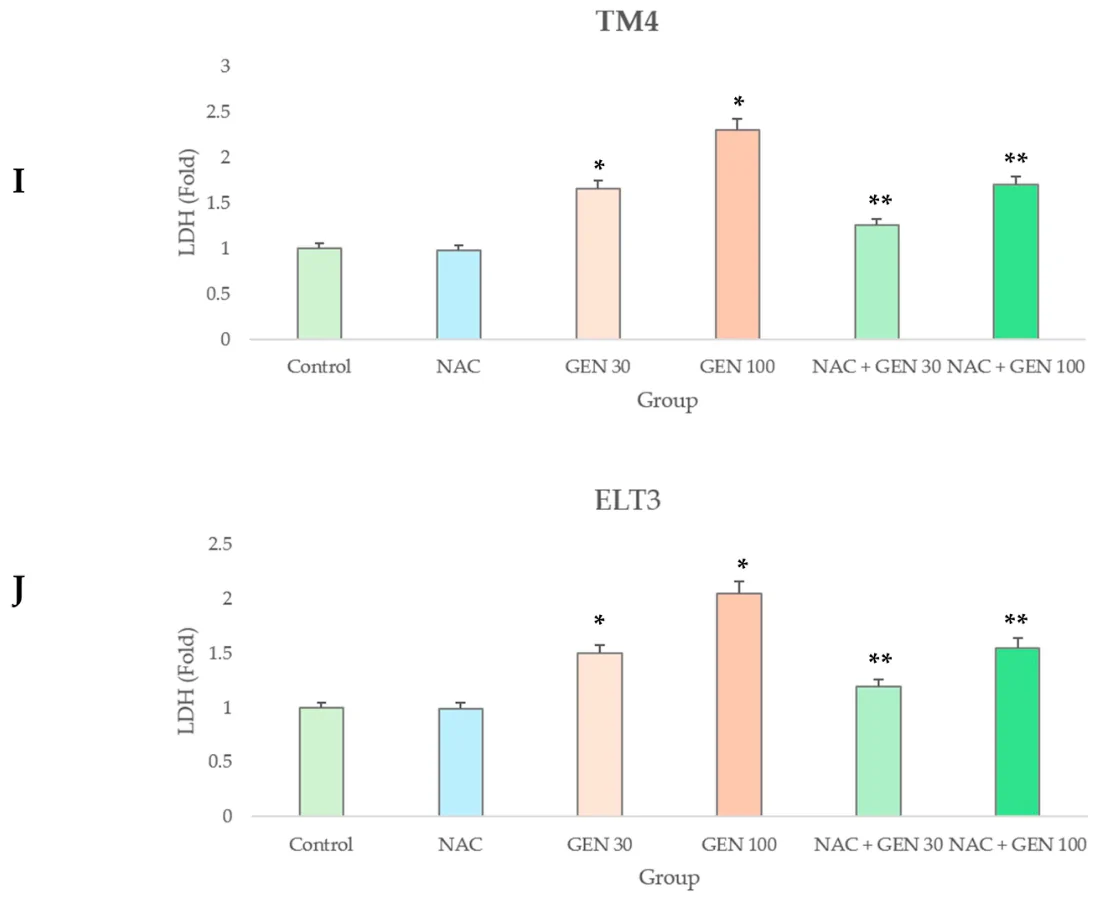
NAC attenuates GEN-induced cell viability and oxidative stress markers in TM4 and ELT3 cells. TM4 and ELT3 cells were pretreated with NAC prior to GEN exposure (30 and 100 µM) for 48 h. The protective effects of NAC were evaluated by measuring (**A**) TM4-cell viability, (**B**) ELT3- cell viability, (**C**) TM4-ROS production, (**D**) ELT3-ROS production, (**E**) TM4-MDA levels, (**F**) ELT3-MDA levels, (**G**) TM4-GR activity, (**H**) ELT3-Gr activity, (**I**) TM4-LDH release, and (**J**) ELT3-LDH release. Data are presented as mean ± SD). Statistical significance was determined by control and GEN-only group (* *p* < 0.05 vs. control; ** *p* < 0.05 vs. GEN alone).

To investigate whether oxidative stress mediates GEN-induced cytotoxicity, TM4 and ELT3 cells were pretreated with the antioxidant NAC prior to GEN exposure. A series of biochemical and cellular parameters related to oxidative damage and cell injury were evaluated. GEN significantly reduced cell viability in both TM4 and ELT3 cells in a concentration-dependent manner. NAC pretreatment significantly attenuated GEN-induced cytotoxicity, partially restoring cell viability in both cell lines (Figure 4A,B). Consistent with the reduction in viability, GEN exposure also resulted in a significant increase in intracellular ROS levels in TM4 and ELT3 cells. ROS generation was significantly elevated in a concentration-dependent manner following GEN treatment, whereas NAC pretreatment markedly suppressed ROS accumulation (Figure 4C,D). In parallel with ROS elevation, GEN significantly increased MDA levels. Pretreatment with NAC significantly reduced MDA levels compared with GEN-treated cells, indicating attenuation of oxidative membrane damage (Figure 4E,F). In contrast, GEN significantly decreased GR activity in both TM4 and ELT3 cells (Figure 4G,H), suggesting impairment of the cellular antioxidant defense system. NAC pretreatment partially restored GR activity, while GEN markedly increased ROS production, indicating enhanced membrane damage and cytotoxicity. NAC pretreatment significantly reduced LDH release compared with the corresponding GEN-treated groups (Figure 4I,J).

### 3.5. NAC Attenuates GEN-Induced Mitochondrial and Energy Markers in TM4 and ELT3 Cells

**Figure 5:**
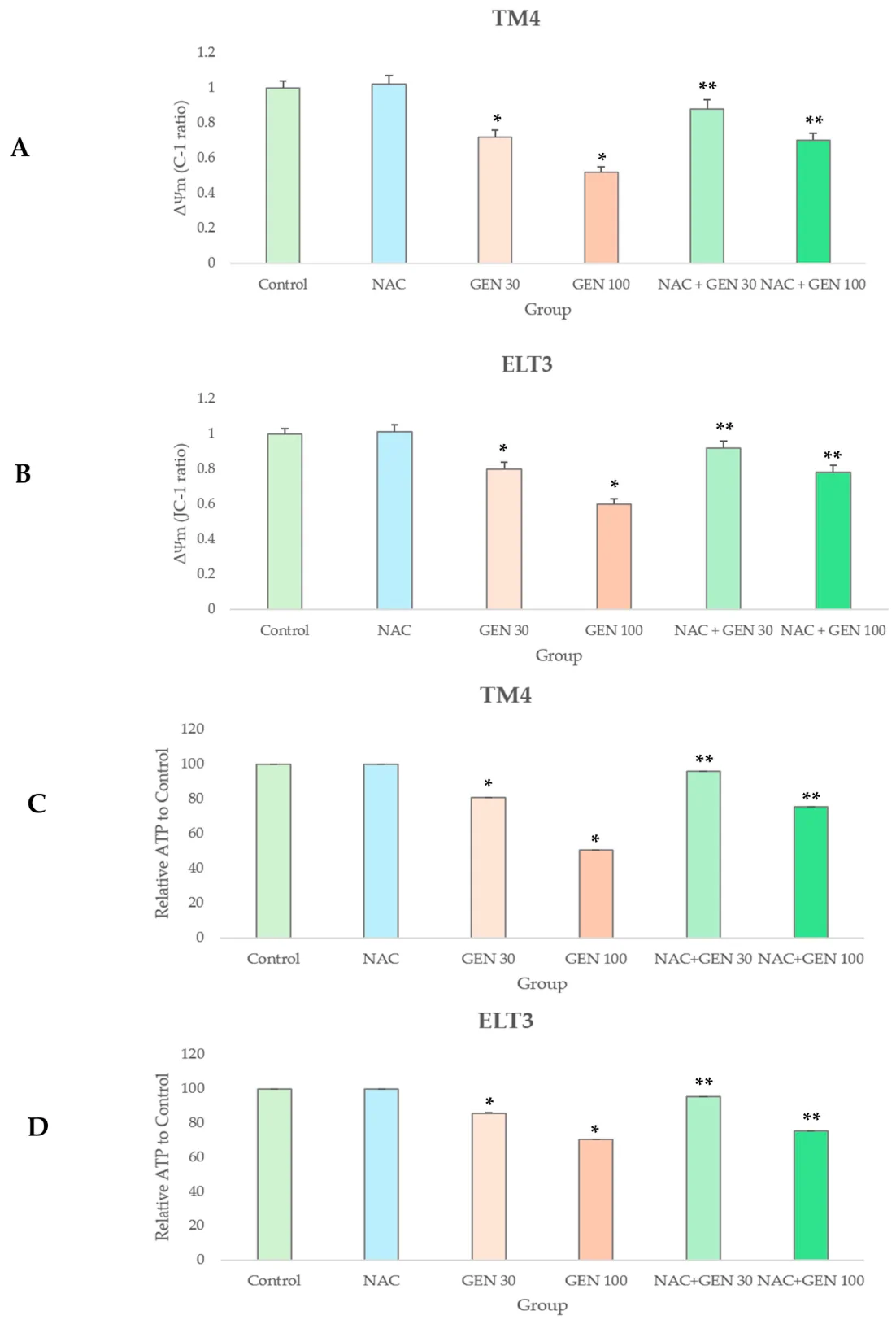
NAC attenuates GEN-induced mitochondrial and bioenergetic alterations in TM4 and ELT3 cells. TM4 and ELT3 cells were pretreated with NAC prior to GEN exposure (30 and 100 µM) for 48 h. The protective effects of NAC were evaluated by measuring (**A**) TM4-ΔΨm, (**B**) ELT3-ΔΨm, (**C**) TM4-ATP levels, and (**D**) ELT3-ATP levels. ATP luminescence was normalized to the total protein content of the corresponding samples and expressed as RLU/mg protein. Data are presented as mean ± SD (n = 3). Statistical significance was determined by comparison with control and GEN-only groups (* *p* < 0.05 vs. control; ** *p* < 0.05 vs. GEN alone).

To investigate whether oxidative stress contributes to GEN-induced mitochondrial and bioenergetic alterations, TM4 and ELT3 cells were pretreated with NAC before GEN exposure. GEN treatment significantly reduced ΔΨm, whereas NAC pretreatment significantly attenuated this reduction in both cell lines (Figure 5A,B). GEN also significantly decreased intracellular ATP levels normalized to total protein content, consistent with impaired cellular bioenergetic function. NAC pretreatment significantly attenuated the GEN-induced reduction in normalized ATP levels (Figure 5C,D). Collectively, these findings support the involvement of oxidative stress in GEN-associated mitochondrial and bioenergetic impairment and indicate that NAC partially protects both TM4 and ELT3 cells against these alterations.

### 3.6. GEN Regulates Apoptosis-Related Gene Expression in TM4 and ELT3 Cells Through Oxidative Stress-Dependent Pathways

**Figure 6:**
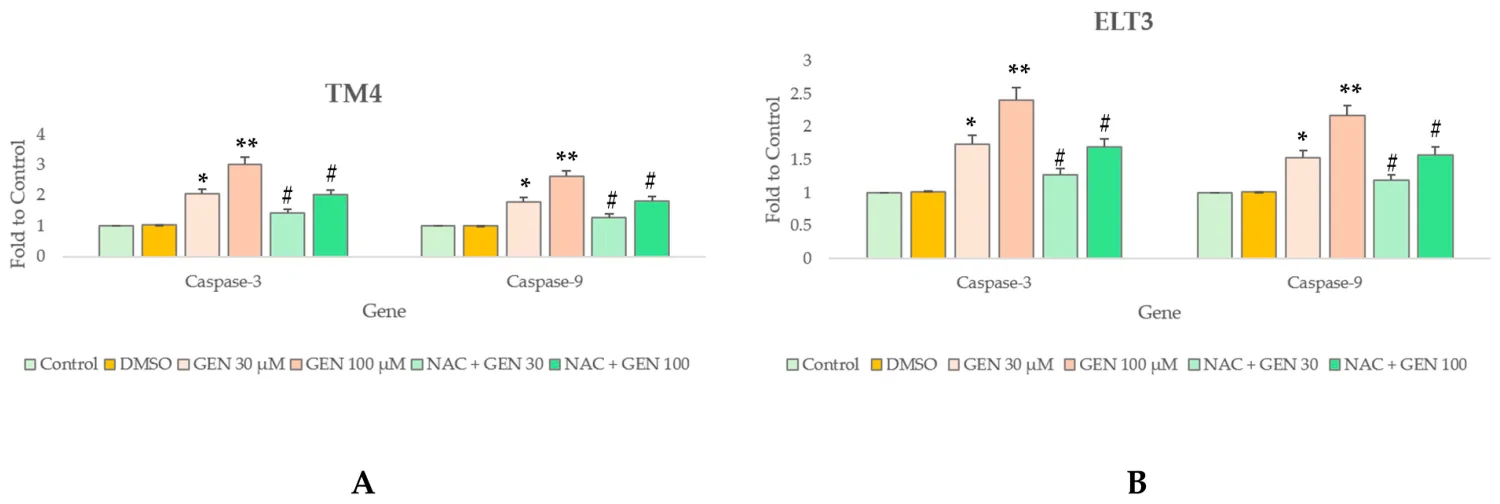

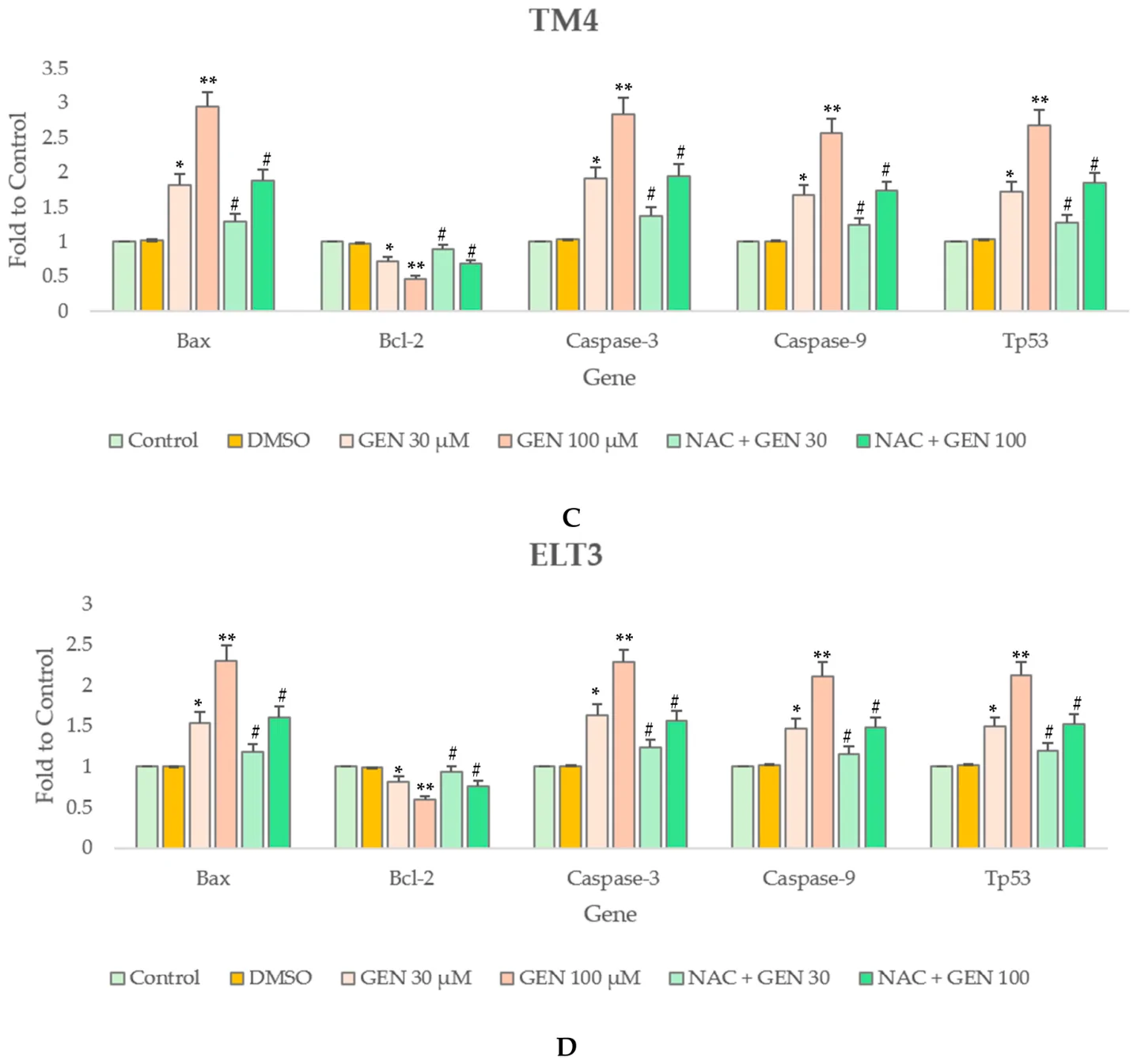
Effects of GEN on caspase activation and apoptosis-related gene expression in TM4 and ELT3 cells through oxidative stress-dependent mechanisms. TM4 cells (**A**,**C**) and ELT3 cells (**B**,**D**) were treated with GEN (30 and 100 µM) in the presence or absence of NAC. Caspase-3 and caspase-9 activities were measured to evaluate activation of the intrinsic apoptotic pathway and expressed as fold change relative to control. Apoptosis-related gene expressions were analyzed by qPCR, including Bax, Bcl-2, caspase-3, caspase-9, and Tp53, and normalized to an internal reference gene before being expressed as fold change relative to control. NAC pretreatment significantly attenuated GEN-induced caspase activation and transcriptional alterations, as evidenced by reduced expression of pro-apoptotic genes and partial restoration of Bcl-2 expression, indicating that oxidative stress plays a critical upstream role in regulating apoptosis-related signaling. Data are presented as mean ± SD from three independent experiments. Statistical significance was determined by comparison with control and GEN-treated groups (* *p* < 0.05 and ** *p* < 0.01 versus the control group; # *p* < 0.05 versus the corresponding GEN-treated group).

Caspase-3 and caspase-9 activities were measured to evaluate apoptosis induction following GEN treatment in TM4 and ELT3 cells (Figure 5). GEN treatment significantly increased caspase-3 and caspase-9 activities compared with the control and DMSO-treated groups in both TM4 (Figure 6A) and ELT3 cells (Figure 6B). No significant difference was observed between the control and DMSO groups, indicating that the solvent had no detectable effect on caspase activity. The increases in caspase activity were concentration-dependent, suggesting activation of the intrinsic apoptotic pathway. Importantly, NAC pretreatment significantly attenuated GEN-induced caspase activation in both cell types. Collectively, these findings demonstrate that GEN induces apoptosis through caspase-dependent mechanisms in TM4 and ELT3 cells, while antioxidant pretreatment with NAC significantly reduces these effects. These results further support oxidative stress as a key upstream regulator of GEN-induced apoptotic signaling. To further investigate the molecular mechanisms underlying GEN-induced cytotoxicity, the mRNA expression levels of apoptosis-related genes were analyzed. GEN treatment significantly increased the expression of Bax, caspase-3, caspase-9, and Tp53, while significantly decreasing Bcl-2 expression compared with the control and DMSO-treated groups in both TM4 (Figure 6C) and ELT3 cells (Figure 6D). No significant differences were observed between the control and DMSO groups. These findings indicate that GEN alters apoptosis-related gene expression in a concentration-dependent manner, consistent with the activation of mitochondrial apoptotic signaling. NAC pretreatment significantly reversed GEN-induced transcriptional alterations by suppressing pro-apoptotic gene expression and partially restoring Bcl-2 levels. Overall, these results demonstrate that GEN modulates apoptosis-related gene expression in TM4 and ELT3 cells through oxidative stress-mediated mechanisms, while NAC significantly mitigates these molecular changes. Together, these findings further support oxidative stress as an upstream trigger of mitochondrial apoptotic signaling. GAPDH raw Cq values remained stable across the control, GEN 30 µM, and GEN 100 µM groups. No significant treatment-associated differences were detected in either TM4 cells (F(2,6) = 0.60, *p* = 0.579) or ELT3 cells (F(2,6) = 0.53, *p* = 0.615; Supplementary Table S1).

## 4. Discussion

The present study demonstrates that GEN induces cytotoxicity in TM4 and ELT3 cells through an oxidative stress-mediated mitochondrial pathway. The integrated findings from cell viability, oxidative stress markers, antioxidant enzyme activity, ΔΨm, ATP content, membrane integrity, caspase activities, and apoptosis-related gene expression support a mechanistic cascade in which impaired antioxidant defense promotes redox imbalance, mitochondrial injury, and subsequent apoptotic signaling.

One of the earliest alterations observed after GEN exposure was suppression of GR activity, indicating impairment of cellular antioxidant defense. GR maintains intracellular redox balance by catalyzing the NADPH-dependent regeneration of reduced glutathione (GSH) from oxidized glutathione (GSSG) [16]. Because GSH is essential for detoxifying ROS and limiting oxidative injury [31], reduced GR activity may weaken antioxidant capacity and amplify ROS accumulation. Disruption of glutathione homeostasis is widely recognized as an initiating event in oxidative stress-mediated toxicity [32]. Consistent with this mechanism, GEN significantly increased intracellular ROS production in both cell types. Excess ROS can damage lipids, proteins, and DNA, thereby contributing to cellular injury and death [33]. Mitochondria are both a major source and a principal target of ROS; oxidative damage to mitochondrial membranes and electron-transport-chain components can further increase ROS generation, creating a self-reinforcing cycle of redox dysfunction [34]. GEN also upregulates MDA, a well-established marker of lipid peroxidation [35]. This finding indicates oxidative damage to membrane polyunsaturated fatty acids and is consistent with the membrane and mitochondrial injury reported in other oxidative-toxicant models [36]. The decrease in GR activity may reflect broader dysregulation of the antioxidant network rather than an isolated enzymatic change. Antioxidant enzymes involved in glutathione synthesis and recycling are regulated, at least in part, by nuclear factor erythroid 2-related factor 2 (Nrf2), which promotes cytoprotective genes such as heme oxygenase-1 (HO-1). Impaired Nrf2-dependent antioxidant signaling could therefore contribute to reduced GR activity and ROS accumulation. However, because Nrf2 activation, HO-1 expression, and glutathione status were not directly measured, this explanation remains a plausible hypothesis rather than a demonstrated mechanism. This caution is particularly important because GEN may exert antioxidant or pro-oxidant effects depending on concentration, exposure duration, and cellular context [37].

Mitochondria are especially vulnerable to oxidative damage because of their high oxygen consumption and proximity to intracellular ROS-generation sites [38]. In both TM4 and ELT3 cells, GEN caused a significant loss of ΔΨm. Collapse of ΔΨm reflects disruption of the electrochemical gradient required for oxidative phosphorylation and is a hallmark of mitochondrial dysfunction [39]; it is also closely associated with mitochondrial permeability changes and activation of the intrinsic apoptotic pathway [40]. Consistent with mitochondrial depolarization, GEN reduced the total intracellular ATP content in both cell types. ATP depletion may compromise ion homeostasis, biosynthesis, and other energy-dependent survival processes [41]. Increased LDH release further indicated loss of plasma-membrane integrity and was consistent with the concurrent reduction in cell viability [22]. Nevertheless, the ATP finding requires cautious interpretation because the luminescence signal was not normalized to total protein or viable cell number. Because GEN reduced viability under the same conditions, part of the lower ATP signal may reflect fewer metabolically active cells rather than selective inhibition of ATP production in surviving cells. Thus, ATP depletion provides supportive, but not definitive, evidence of bioenergetic dysfunction; mitochondrial involvement is more strongly supported by the concurrent loss of ΔΨm, oxidative injury, intrinsic caspase activation, and NAC-mediated protection [41].

Pretreatment with NAC substantially attenuated GEN-induced cellular injury. NAC reduced ROS accumulation and lipid peroxidation, restored GR activity, preserved ΔΨm, and partially restored ATP content. As a precursor for intracellular GSH synthesis and a ROS scavenger [42], NAC can reinforce cellular antioxidant capacity and interrupt ROS-dependent injury; its protective effects therefore provide functional evidence that oxidative stress acts upstream of mitochondrial dysfunction in the pathway induced by GEN. Similar antioxidant-mediated preservation of mitochondrial function has been described in other oxidative stress toxicology models [43]. The concentrations used in this study were selected to characterize mechanistic responses under elevated, acute exposure conditions, rather than to reproduce normal dietary exposure [28]. Flavonoids and isoflavones may exhibit concentration-dependent dual effects, acting as antioxidants at lower concentrations but becoming pro-oxidant and cytotoxic at higher concentrations [44,45]. Accordingly, the coordinated GR suppression, ROS accumulation, lipid peroxidation, mitochondrial depolarization, ATP reduction, and NAC rescue observed here are consistent with a high-concentration pro-oxidant response. Other upstream pathways may also contribute; for example, GEN-induced apoptosis in prostate cancer cells was preceded by reduced focal adhesion kinase activity, suggesting that disruption of adhesion- and survival-related signaling may occur before the execution of apoptosis [46]. These potential upstream mechanisms require direct testing in TM4 and ELT3 cells. Importantly, the reduction in intracellular ATP remained evident after normalization to total protein content, indicating that the decrease was not solely attributable to the loss of cellular material following GEN exposure. When considered together with the reduction in ΔΨm, these findings support impaired cellular bioenergetic function associated with mitochondrial injury. The greater reduction in normalized ATP observed in TM4 cells is also consistent with their markedly higher sensitivity to GEN compared with ELT3 cells. Nevertheless, intracellular ATP content is influenced by both mitochondrial oxidative phosphorylation and glycolytic metabolism. Therefore, the present findings should be interpreted as evidence consistent with bioenergetic impairment rather than direct confirmation of defective mitochondrial respiration. Future studies examining oxygen-consumption rate and extracellular acidification rate would help distinguish mitochondrial and glycolytic contributions to GEN-induced ATP depletion.

GEN-induced mitochondrial dysfunction was accompanied by activation of the intrinsic apoptotic pathway. Caspase activity assays showed significant increases in caspase-9 and caspase-3 in both cell lines. Caspase-9 is activated after mitochondrial outer-membrane permeabilization and cytochrome c release [40] and subsequently activates executioner caspases, including caspase-3, which mediate proteolytic dismantling of the cell [47]. The attenuation of both caspase activities by NAC further indicates that ROS generation occurs upstream of caspase-dependent apoptosis. At the transcriptional level, GEN increased the expression of Bax, caspase-3, caspase-9, and Tp53 while decreasing Bcl-2 expression. The Bax/Bcl-2 balance is a critical determinant of mitochondrial integrity: Bax promotes mitochondrial outer-membrane permeabilization, whereas Bcl-2 stabilizes the membrane and suppresses apoptosis [48]. Upregulation of Tp53 is also consistent with the activation of a cellular stress-response program, because Tp53 regulates Bax and other apoptosis-related genes in response to oxidative stress and DNA damage [49,50]. Although oxidative DNA damage and cytochrome c release were not directly evaluated, the coordinated increases in ROS, Tp53, Bax, caspase-9, and caspase-3, together with Bcl-2 downregulation and ΔΨm loss, support a mechanistic sequence in which impaired glutathione recycling promotes ROS accumulation, oxidative membrane injury, mitochondrial permeabilization, and caspase-dependent apoptosis [32,33,38,48,49,50]. These convergent findings are consistent with established models of ROS-regulated mitochondrial apoptosis [39,51]. However, because the present molecular evidence is based on gene expression and enzyme-activity assays, the proposed sequence should be interpreted as a supported mechanistic model rather than as proof of every intermediate step.

Although TM4 and ELT3 cells showed a broadly similar pattern of oxidative stress, mitochondrial impairment, and apoptosis, their quantitative sensitivities differed markedly. At 48 h, the GEN IC50 was 30.1 µM in TM4 cells and 132.5 µM in ELT3 cells; thus, TM4 cells were approximately 4.4-fold more sensitive. Sertoli cells support spermatogenesis and require tightly regulated redox homeostasis to maintain testicular function [51]; their greater susceptibility therefore raises a potential reproductive toxicological concern under elevated exposure conditions. ELT3 cells are tumor-derived uterine leiomyoma cells and may differ from TM4 cells in antioxidant capacity, mitochondrial metabolism, proliferative state, transporter expression, and stress-response signaling; these features could contribute to their lower sensitivity, although they were not examined directly. Importantly, the present findings do not demonstrate preferential toxicity toward leiomyoma cells or establish a favorable therapeutic window. Instead, the normal Sertoli-cell model was affected at substantially lower concentrations than the tumor-derived model. The apoptotic response in ELT3 cells should therefore be described as concentration-dependent cytotoxicity with possible antitumor relevance, not as evidence of selective antitumor activity [51].

Several limitations should be acknowledged. First, the GEN concentrations examined (30–100 µM) exceeded the circulating concentrations generally achieved after dietary soy consumption, which typically remain within the sub-micromolar to low-micromolar range and consist predominantly of conjugated metabolites [52,53]. The experimental conditions therefore represent an acute mechanistic toxicology model of elevated GEN exposure rather than physiological nutritional exposure. Furthermore, the use of only two immortalized cell lines cannot reproduce tissue complexity, systemic metabolism, endocrine regulation, tissue distribution, or chronic exposure dynamics in vivo. Consequently, the results should not be directly extrapolated to human reproductive toxicity or therapeutic efficacy. Second, both cell lines were maintained in the same medium to permit comparison under standardized conditions, but the effects of alternative culture conditions on basal ELT3 metabolism and redox status were not examined. An influence of medium composition on cellular responsiveness therefore cannot be ruled out. In addition, ΔΨm was assessed using the JC-1 red/green fluorescence ratio without complementary microscopy or flow cytometry. Although intracellular ATP levels were normalized to total protein content in the revised analysis, other plate-based measurements, including LDH release, intracellular ROS, MDA, and GR activity, may still have been influenced by treatment-related differences in viable cell number because assay-specific normalization was not performed [54]. These findings should therefore be interpreted cautiously. Future studies should incorporate appropriate normalization to viable cell number or total protein content, direct measurements of mitochondrial respiration, and complementary imaging or flow-cytometric analyses. Third, qPCR analysis demonstrated transcriptional regulation but did not necessarily indicate corresponding changes in protein abundance. Although the coordinated changes in apoptosis-related gene expression, caspase-3 and caspase-9 activities, mitochondrial function, oxidative stress markers, and the protective effects of NAC provide convergent evidence supporting the involvement of ROS-associated intrinsic apoptotic signaling, the proposed mechanism cannot be considered fully established without direct protein-level confirmation. Further studies using Western blotting or other quantitative protein assays are therefore required to validate changes in Bax, Bcl-2, Tp53, and the cleaved forms of caspase-3 and caspase-9. Although raw Cq analysis supported the stability of GAPDH across the present treatment conditions, its use as the sole reference gene remains a methodological limitation. Future studies should validate multiple candidate reference genes and employ the most stable reference gene or the geometric mean of multiple validated reference genes, in accordance with the MIQE recommendations [47,55,56]. Finally, apoptotic markers were measured only at 48 h; the data therefore represent a single endpoint rather than the complete temporal sequence, and earlier caspase activation or progression toward secondary necrosis cannot be ruled out. Overall, the findings provide mechanistic evidence that elevated GEN exposure induces a redox-sensitive mitochondrial apoptotic response under controlled in vitro conditions. The greater sensitivity of TM4 cells highlights a potential reproductive toxicological concern, whereas apoptosis in ELT3 cells indicates cytotoxic responsiveness but does not establish selective antitumor efficacy. Studies using physiologically relevant concentrations, time-course designs, primary cells, three-dimensional cultures, validated molecular markers, pharmacokinetic approaches, and in vivo models are required before reproductive risk or therapeutic benefit can be inferred.

## 5. Conclusions

The present study demonstrates that elevated GEN exposure induces oxidative stress-mediated mitochondrial injury and intrinsic apoptotic signaling in TM4 and ELT3 cells. Suppression of glutathione reductase activity was accompanied by ROS accumulation and lipid peroxidation, followed by mitochondrial membrane depolarization, reduced ATP content, activation of caspase-9 and caspase-3, upregulation of Tp53 and Bax, and downregulation of Bcl-2. The protective effects of NAC provide functional evidence that oxidative stress acts upstream of mitochondrial dysfunction and caspase-dependent apoptosis.

Importantly, TM4 Sertoli cells were approximately 4.4-fold more sensitive to GEN than ELT3 leiomyoma cells at 48 h. Therefore, the findings do not demonstrate preferential cytotoxicity toward leiomyoma cells or establish a favorable therapeutic window under the present experimental conditions. Instead, the marked susceptibility of TM4 cells raises a potential reproductive toxicological concern associated with elevated GEN exposure. Because the concentrations examined exceed those typically achieved through dietary intake, these findings should be interpreted as mechanistic evidence obtained from an acute in vitro toxicological model rather than as direct evidence of human reproductive toxicity or therapeutic efficacy. Further studies using physiologically relevant concentrations, time-course designs, primary or three-dimensional cell models, protein-level validation, and in vivo approaches are needed in order to determine the broader biological and translational significance of these findings.

## Figures and Tables

**Table 1:** Species-specific primers used for qPCR analysis in TM4 and ELT3 cells.

Cell Line	Gene	Reference Sequences	Forward (5′-3′)	Reverse (5′-3′)	Amplicon Size (bp)
Mouse	Tp53	NM_011640.3	AGCTCCTCTCCCAGCTTGAA	TGTGCAGGTTCTTGGAGTCA	132
Mouse	Bcl-2	NM_009741	CCTGTGGATGACTGAGTACCTG	AGCCAGGAGAAATCAAACAGAGG	120
Mouse	Bax	NM_007527	AGGATGCGTCCACCAAGAAGCT	TCCGTGTCCACGTCAGCAATCA	115
Mouse	Caspase-3	NM_009810	GGAGTCTGACTGGAAAGCCGAA	CTTCTGGCAAGCCATCTCCTCA	130
Mouse	Caspase-9	NM_015733	GCTGTGTCAAGTTTGCCTACCC	CCAGAATGCCATCCAAGGTCTC	140
Mouse	GAPDH	NM_008084	CATCACTGCCACCCAGAAGACTG	ATGCCAGTGAGCTTCCCGTTCAG	120
Rat	Tp53	NM_030989	TCTCCCCAGCAAAAGAAAAA	TTTTATGGCGGGACGTAGAC	170
Rat	Bcl-2	NM_016993	GGGATGCCTTTGTGGAACTA	CATATTTGTTTGGGGCAGGT	200
Rat	Bax	NM_017059	AAAGACATTGGAGCCACCAC	TATTGCCTGCCACAAACTCA	150
Rat	Caspase-3	NM_012922	AGGGGCATGTTTCTGTTTTG	CATTGCAGGCAGTGGTATTG	150
Rat	Caspase-9	NM_031632	TCATTCTTGCAAAGCAGTGG	TGGGTGTTTCTGGTGTGAGA	200
Rat	GAPDH	NM_017008.4	ATGGGAGCTGGTCATCAAC	GTGGTTCACACCCATCACAA	223

## Data Availability

The original contributions presented in this study are included in the article/Supplementary Materials. Further inquiries can be directed to the corresponding author.

## Supplementary Materials

The following supporting information can be downloaded at: https://www.mdpi.com/article/10.3390/jox16050156/s1, Table S1: Stability assessment of GAPDH raw Cq values in TM4 and ELT3 cells following GEN exposure.

## Abbreviations

The following abbreviations are used in this manuscript:

GEN	Genistein
LDH	Lactate dehydrogenase
ROS	Reactive oxygen species
MDA	Malondialdehyde
GR	Glutathione reductase
ΔΨm	Mitochondrial membrane potential
qPCR	Quantitative real-time PCR
NAC	N-acetylcysteine
ELI3	Eker leiomyoma tumor-3 cells
TM4	Mouse Sertoli cells
DMSO	Dimethyl sulfoxide
pNA	p-nitroaniline
