# Supplementary material for: Oxidative Stress-Mediated Mitochondrial Dysfunction Drives Genistein-Induced Apoptosis in TM4 Sertoli and ELT3 Leiomyoma Cells
Source: J Xenobiot. 2026 Aug 22;16(5):156. doi: 10.3390/jox16050156 (PMC13608690; doi:10.3390/jox16050156)
Supplement: Supplementary file 1 [file jox-16-00156-s001.zip › jox-4492800-supplementary.pdf]

Supplementary Materials: Oxidative Stress–Mediated Mitochondrial Dysfunction Drives Genistein-Induced Apoptosis in TM4 Sertoli and ELT3 Leiomyoma Cells

Samak Sutjarit , Chainarong Sakulthaew, Nattakan Meekhanon and Kazuhiko Ochiai

Table S1. Stability assessment of GAPDH raw Cq values in TM4 and ELT3 cells following GEN exposure. Data are expressed as mean ± SD from three independent experiments. Statistical differences were analyzed separately for each cell line using one-way ANOVA followed by Tukey's multiple-comparisons test. No significant differences were detected among the treatment groups ($p > 0.05$).

Cell line	Group	GAPDH Cq, mean ± SD	CV
TM4	Control	20.317 ± 0.015	0.08%
TM4	GEN 30 μM	20.307 ± 0.006	0.03%
TM4	GEN 100 μM	20.317 ± 0.015	0.08%
ELT3	Control	20.347 ± 0.035	0.17%
ELT3	GEN 30 μM	20.360 ± 0.026	0.13%
ELT3	GEN 100 μM	20.370 ± 0.020	0.10%
